# Overview of Anti-SARS-CoV-2 Immune Response Six Months after BNT162b2 mRNA Vaccine

**DOI:** 10.3390/vaccines10020171

**Published:** 2022-01-22

**Authors:** Claudia Gandolfo, Gabriele Anichini, Marco Mugnaini, Monica Bocchia, Chiara Terrosi, Anna Sicuranza, Gianni Gori Savellini, Alessandro Gozzetti, Federico Franchi, Maria Grazia Cusi

**Affiliations:** 1 Virology Unit, Department of Medical Biotechnologies, University of Siena, Azienda, Ospedaliera Universitaria Senese, V. le Bracci, 16, 53100 Siena, Italy; claudia.gandolfo@unisi.it (C.G.); gabriele.anichini@student.unisi.it (G.A.); chiara.terrosi@unisi.it (C.T.); gianni.gori@unisi.it (G.G.S.); 2 Department of Information Engineering and Mathematical Sciences, University of Siena, 53100 Siena, Italy; marco.mugnaini@unisi.it; 3 Hematology Unit, Department of Medical Sciences, Surgery and Neurosciences, Azienda, Ospedaliera Universitaria Senese, V. le Bracci, 16, 53100 Siena, Italy; monica.bocchia@unisi.it (M.B.); sicuranza4@unisi.it (A.S.); gozzetti@unisi.it (A.G.); 4 Anesthesia and Intensive Care Unit, Department of Medicine, Surgery and Neuroscience, University of Siena, 53100 Siena, Italy; federico.franchi@unisi.it

**Keywords:** SARS-CoV-2, BNT162b2 mRNA vaccine, humoral immunity, cellular immunity, fuzzy system

## Abstract

Background: We have designed a prospective study aiming to monitor the immune response in 178 health care workers six months after BNT162b2 mRNA vaccination. Methods: The humoral immune response of all subjects was evaluated by chemiluminescence (CMIA); in 60 serum samples, a live virus-based neutralization assay was also tested. Moreover, 6 months after vaccination, B- and T-cell subsets from 20 subjects were observed by FACS analysis after restimulation with the trimeric SARS-CoV-2 Spike protein as an antigen, thus mimicking reinfection in vitro. Results: A significant decrease of circulating IgG levels and neutralizing antibodies over time were observed. Moreover, six months after vaccination, a variable T-cell immune response after in vitro antigen stimulation of PBMC was observed. On the contrary, the analysis of B-cell response showed a shift from unswitched to switched memory B-cells and an increase of Th17 cells. Conclusions: Although the variability of the CD4^+^ and CD8^+^ immune response and an antibody decline was observed among vaccinated subjects, the increase of switched memory B-cells and Th17 cells, correlating with the presence of neutralizing antibodies, opened the debate on the correct timing of vaccination.

## 1. Introduction

After the global diffusion of the severe acute respiratory infectious disease caused by the SARS-CoV-2 virus (COVID-19) in 2020, the World Health Organization (WHO) declared a pandemic status. Up to now, millions of COVID-19 cases have been confirmed worldwide (WHO, 2021) [1]. Considering its rapid spread, pharmaceutical industries promptly started intensive work to develop specific and efficacious vaccines, thanks to government support. The BNT162b2 mRNA vaccine (Pfizer-BioNTech) was the first vaccine available in Italy to prevent COVID-19 [2,3]. BNT162b2 is a lipid nanoparticle formulated nucleoside-modified messenger RNA (mRNA), encoding SARS-CoV-2 spike (S) protein, stabilized in the prefusion conformation [4]. Results from clinical trials showed that up to 6 months of follow-up and despite a gradually declining trend in vaccine efficacy, BNT162b2 had a favorable safety profile and a 91.3% effectiveness against COVID-19 disease [3].

However, on the basis of published results, 10–22% of people immunized against COVID-19, showing a steady decline of the humoral response [5], could present a major risk of breakthrough infection, particularly with variants [6]. Many studies have been conducted on the duration of immunity after natural infection by SARS-CoV-2 [7], and results have been produced concerning the persistence of the antibody response over time and the durability of the cell-mediated immune response, in particular memory B and T cells, after two doses of vaccine [6,8,9,10].

Therefore, we designed a prospective study (MOTIVE study), (aiming) to explore and monitor the humoral immune response induced by the BNT162b2 mRNA vaccine in 178 vaccinated volunteers, (ii) to examine the memory B- and T-cell responses after the second BNT162b2 mRNA vaccine boost, and (iii) to run predictor models for the presence of a good protective antibody response against SARS-CoV-2. It is of primary interest to know the real correlates of protection from COVID-19 vaccines therefore a deeper knowledge of the type of immunity and its duration after vaccination could help to understand whether and when it is necessary to initiate further jabs and improve the vaccine performance against variants.

## 2. Materials and Methods

### 2.1. Study Design and Participants

In this current observational cohort study, we enrolled 178 volunteers among healthcare workers (HCWs): 61 males and 117 females from ‘Santa Maria alle Scotte’ University Hospital in Siena, who had been subjected to periodical control (every 2 weeks) by molecular testing for SARS-CoV-2 virus with a nasopharyngeal swab and had never been infected. All subjects were vaccinated with two doses of BNT162b2 mRNA Vaccine (Pfizer Inc., New York, NY, USA) between 27 December 2020 and 31 January 2021. Among them, only 53 randomly selected subjects were screened 10 days after receiving the first dose of the vaccine. Then, blood samples were drawn to all subjects 10 days, one, three, and six months after the second dose of the BNT162b2 mRNA vaccine for humoral response analysis. Cell-mediated immune response was also investigated six months after vaccination in 20 vaccinated subjects, randomly selected for their high (10 subjects, neutralizing antibody titer one month after vaccination >64) or low (10 subjects, neutralizing antibody titer one month after vaccination <64) antibody response.

All subjects gave their informed consent to participate in this study in accordance with the principles of the Declaration of Helsinki. The study was approved by the local Ethical Committee (ID 19290).

### 2.2. Anti-SARS-CoV-2 Spike IgG Antibodies

In order to evaluate the humoral response induced by the vaccine, a blood sample was drawn from all HCWs 10 days, 1, 3, and 6 months after the second vaccine administration. To this aim, whole blood samples were collected and centrifuged at 1600 *g* for 15 min to separate the serum. Then they were stored at −20 °C until serological assays were performed. Subjects’ sera were analyzed using an Abbott SARS-CoV-2 IgG II Quant assay (Abbott Laboratories, Chicago, IL, USA), a chemiluminescent microparticle immunoassay (CMIA) for evaluating the immune status of individuals with quantitative measurement of IgG antibodies against the spike receptor-binding domain (RBD) of SARS-CoV-2. This assay was performed on an Abbott Architect i2000 (Abbott Diagnostics), according to the manufacturer’s instructions. The cut-off value was 50.00 AU/mL. A sample was considered positive when the result was >50.0 AU/mL.

### 2.3. Microneutralization Assay

SARS-CoV-2 virus neutralization assay was carried out on Vero E6 cells (ATCC^®^ CRL-1586™) in a 96-well microplate (COSTAR, Corning Incorporated, Corning, NY, USA). Twenty-five microliters of two-fold serial dilutions (1:8 to 1:1024) of sera samples were added to an equal volume of the SARS-CoV-2 strain (SARS-CoV-2/human/ITA/Siena-1/2020; GenBank: MT531537.2), containing 100 TCID_50_ and incubated for 90 min at 37 °C. Finally, 50 μL of Vero E6 cells suspension (2 × 10^5^ cells/mL), prepared in complete DMEM, was added to each well. After 72 h of incubation at 37 °C, cultures were daily examined for the presence of CPE under a microscope (Olympus IX51). The 50% endpoint titer was calculated using the Reed–Muench method [11]. A positive and negative control serum was included in each assay. Geometric mean titers (GMTs) of the neutralization assays were calculated. Serum from the National Institute for Biological Standards and Control, Blanche Lane, Ridge, Herts, UK (NIBSC) with known neutralization titer (Research reagent for anti-SARS-CoV-2 Ab NIBSC code 20/130) was used as a reference in MNTSera of subjects collected before 2019 were used as negative controls.

### 2.4. Peripheral Blood Mononuclear Cells Isolation and Stimulation

For the evaluation of the B- and T-cell response after stimulation with the Spike protein, peripheral blood mononuclear cells (PBMCs) were isolated from 20 selected subjects’ whole blood and three unvaccinated, uninfected negative controls, using Lympholyte^®^ Cell Separation Media (Cedarlane, Burlington, ON, Canada; Cat# DVCL5015). Afterwards, PBMCs were washed with RBC (Red Blood Cell) lysis buffer and seeded in quadruplicate at a concentration of 1 × 10^6^ in 500 µL in 24-well plates in RPMI-1640 medium (Euroclone, Milan, Italy; Cat# ECB2000), supplemented with 10% heat-inactivated human serum (Euroclone, Milan, Italy; Cat# ECS5000L). Thereafter, IL-2 (20 U/mL), IL-10 (50 ng/mL), GM-CSF (50 ng/mL), and IL-4 (0.5 ng/mL) were added to each well and cells were incubated at 37 °C with 5% CO_2_. After 48 h, Trimeric recombinant Spike protein (Leinco Technologies, Inc, St. Louis, MO, USA; Cat# S848) was added in two wells (for B- and T-subsets analysis, respectively) for each subject at a concentration of 5 µg/mL, while the other two wells were kept as unstimulated control cells. T- and B-cell populations were analyzed 24 h and 96 h after antigen stimulation, respectively. Responsiveness of each sample was assessed by stimulation with 5 µg/mL of phytohaemagglutinin (PHA) (Roche Diagnostics, Germany; Cat# 11249738001). After 24 h, cells were harvested and analyzed by flow cytometry.

### 2.5. Multiparameter Flow Cytometry Analysis

T- and B-cell subsets were stimulated in vitro for 24 and 96h with the recombinant Spike, respectively. Afterwards, PBMCs of 20 selected HCWs were harvested and washed, using PBS supplemented with 3% FCS, then stained with a fixable viability dye (LIVE/DEAD™ Fixable Aqua Dead Cell Stain Kit, Thermo Fisher Scientific, Waltham, MA, USA; Cat# L34957) for 20 min at 4 °C.

Surface staining with antibodies binding to CD3 (clone SK7), CD4 (clone SK3), CD8 (clone RPA-T8), CCR7 (clone 150503), CD45RA (clone HI100), CD38 (clone HIT2), HLA-DR (clone G46-6), CXCR3 (clone 1C6) and CCR6 (clone 11A9) for the analysis of T-helper and memory panels and with CD3 (clone SK7), HLA-DR (clone G46-6), CD19 (clone HIB19), CD27 (clone L128), IgD (clone IA6-2), CD20 (clone 2H7), CD24 (clone ML5), and CD38 (clone HIT2) for the analysis of B memory cells panel, according to manufacturer’s instructions. All antibodies were supplied from BD Biosciences (New York, NY, USA). After fixation with PBS + 2% paraformaldehyde for 20 min at 4 °C, cells were washed and resuspended in PBS, supplemented with 0.5 mM EDTA before being acquired with SO LSRFortessa X20 flow cytometer (BD Biosciences, New York, NY, USA). Data analysis was performed using FlowJo v10 (TreeStar, Ashland, OR, USA).

### 2.6. Interferon-γ Quantification

Covi-FERON FIA (IFN-gamma/IFN-γ) (SD, BIOSENSOR), a fluorescent immunoassay, was used to quantify the interferon-γ production in the 20 selected subjects’ whole blood six months after vaccination. Briefly, heparinized whole blood of each subject was incubated O/N at 37 °C in different blood collection tubes, which were antigen-sensitized. These tubes included: SARS-CoV-2 specific proteins tubes, Nil tube (Negative Control) and Mitogen tube (Positive Control). After centrifugation at 2300 g for 15 min, plasma was harvested and tested for the presence of IFN-γ, produced in response to the specific antigens by FIA. Results of each sample were automatically calculated in the analyzer. The concentration of IFN-γ was provided as IU/mL. The test was considered valid if the Nil value was ≤8.0 and the Mitogen value greater than the Nil value by at least 0.5 IU/mL. Results were interpreted as described by the manufacturer’s instructions.

### 2.7. Statistical Analysis

Differences among age, sex, circulating IgG levels, and neutralizing geometric mean titers (GMTs) were evaluated, and statistical significances were assessed with a two-tailed chi-square test. Results were considered statistically significant at *p <* 0.05. For each variable, a 95% confidence interval (95% CI)as calculated and reported. All analyses were performed by using Graph Pad Prism software (v.7.0). To represent data and to perform result comparisons, each single cell population analyzed by cytofluorimetry was normalized to the total amount of cells of interest for the specific subset of the cell population. After normalization, the obtained data were considered in terms of the relative difference between cells that had been non-exposed or exposed to SARS-CoV-2 Spike protein in order to investigate variations in cell counts after one (for T cells) or four days (for B cells) of antigen stimulation. CD4^+^ CM represents the Central memory population, normalized with respect to total CD4^+^ counted cells, CD4^+^ EM are the effector memory population, normalized with respect to total CD4^+^ counted cells as well as Th17 cells. CD8^+^ CM and CD8^+^ EM are the central and effector memory cells normalized for the CD8^+^ population. Finally, the naïve, transitional, memory switched, and unswitched cells were normalized to the overall amount of B-cells for each subset of the cell population. Considering the fuzzy algorithm implemented, the authors used the Fuzzy MATLAB Toolbox and exploited the Mamdani model.

### 2.8. Limitations

This study has some limitations: the in vitro neutralization and stimulation assays were performed only with the WT virus since, at the beginning of this study, it was the only circulating strain.

In addition, another limitation could be represented by the low number of subjects analyzed for the T-cell response, mainly due to the complexity of the protocol used.

## 3. Results

### 3.1. Monitoring of Humoral Response after Vaccination

**Figure 1:**
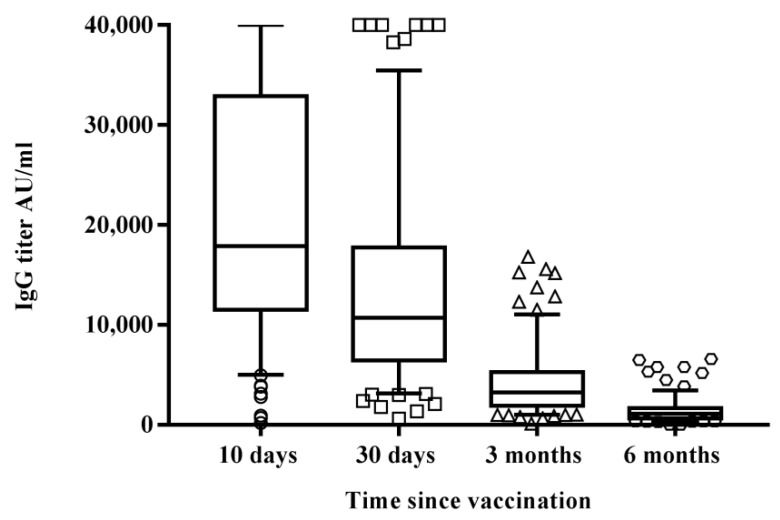
Differences in circulating IgG antibodies between the different time samplings after receiving the second vaccine administration reported as IgG titer in AU/mL. Results are reported in the box-whisker plots as median IgG and upper and lower quartiles. Differences in antibody titers were evaluated, and the statistical significance was assessed using the two-tailed chi-square test. Results were considered statistically significant at *p* < 0.05. Analyses were performed by using Graph Pad Prism software (v.7.0). Among the study group, 60 subjects were screened for the presence of specific neutralizing antibodies against SARS-CoV-2, 10 days, 1, 3, and 6 months after the second vaccine administration. The test was performed by assessing the protective activity of the humoral response against the live SARS-CoV-2 virus. Results did not show a significant difference (*p* = 0.08) in the antibody response, in terms of GMT 10days (GMT = 108.70) or 30 days (GMT = 91.50) after vaccination (Figure 2). However, in the following months, the decline of neutralizing antibodies was consistent, with a significant decrease both three months (GMT = 32.4; *p* < 0.0001 vs. 30 days) and six months (GMT = 17.5; *p* < 0.0001 vs. three months) after the vaccine administration.

We analyzed sera from 178 healthcare workers, 61 males (34.2%; mean age 45.8 years, CI 95% 42.6–49.1) and 117 females (65.8%; mean age 44.4 years, CI 95% 42.1–46.6) who had never been infected by SARS-CoV-2 10 days, one month, three months, and six months after the second dose of BNT162b2 mRNA vaccine (Figure 1). Only two out of 53 randomly selected subjects, who were screened for the presence of specific IgG after the first dose of vaccine, were seronegative (3.8%), while all 178 HCWs were positive 10 days, one month, and three months after the second administration.

Results showed a significant difference in overall titers between 10 (21,394.9 AU/mL; CI 95% 19,600–23,200) and 30 (13,523.1 AU/mL; CI 95% 12,100–14,900), with an evident IgG decrease (36.64%) one month after vaccination (*p* < 0.0001). A similar decreasing pattern of circulating antibodies was observed both three months (4063.2 AU/mL; CI 95% 3600–4530, *p <* 0.0001 compared with results after 30 days) and six months (1431.6 AU/mL; CI 95% 1260–1,600, *p* < 0.0001 compared with results after three months) after the vaccine administration.

Antibody titers decayed over six months but remained detectable in all subjects, but two, who were previously tested positive. These data were confirmed by the decline of neutralizing antibodies.

Finally, to better analyze the declining trend of neutralizing antibodies over time, we assessed the antibody profile of each subject. While those having a GMT > 128 (30 subjects) after 10 days showed a GMT decrease after 30 days (86.7%), the remaining (30 subjects), having a GMT < 128, behaved more heterogeneously. Seven of them (23.3%) showed a reduction in GMT, 17 (56.7%) developed a higher antibody titer, and 6 (20%) remained stable (Table 1).

However, those having a higher GMT soon after vaccination showed a more pronounced decrease of neutralizing antibodies over time, suggesting that a leveling of antibody titer had occurred in all subjects over time (Table 1).

### 3.2. Cell-Mediated Immune Response to SARS-CoV-2 mRNA Vaccine

In addition to the analysis of the specific antibody developed in vaccinated subjects six months after vaccination, we analyzed the B- and T-cell immune responses in 20 subjects. We selected 10 subjects having a low neutralizing antibody response (<64) and 10 having a high antibody response (>64) one month after complete vaccination in order to investigate whether this parameter could correlate with a typical cell-mediated immune response. Three negative controls, represented by subjects who were not infected by SARS-CoV-2 and were not vaccinated, were also included.

We assessed the variation of the percentage of specific B- and T-cell populations between the basal level without antigen stimulation and the one induced by stimulation with the Spike antigen, as described in Section 2.4, in order to mimic the real-life event.

**Figure 3:**
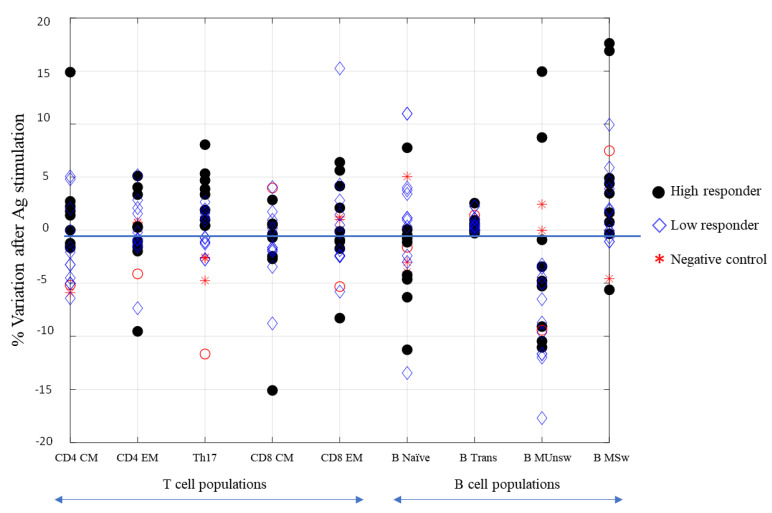
Representation of normalized parameters related to the immune cell populations with respect to their relative variations before and after stimulation with the SARS -CoV-2 Spike protein. High-responding subjects (black circles), low-responding ones (diamonds), and controls (stars). T cell populations: CD4^+^ CM (central memory, CD45RA^−^ CCR7^+^), CD4^+^ EM (effector memory, CD45RA^−^ CCR7^−^), CD4^+^ Th17 (CXCR3^−^ CCR6^+^), CD8^+^ CM (central memory, CD45RA^−^ CCR7^+^), CD8^+^ EM (effector memory, CD45RA^−^ CCR7^−^). B-cell populations: B naïve (CD19^+^ CD27^−^ IgD^+^), B transitional (CD19^+^ CD27^−^ IgD^+^ CD24^high^ CD38^high^), B MUnsw (memory unswitched CD19^+^ CD27^+^ IgD^+^), and B MSw (memory switched CD19^+^ CD27^+^ IgD^−^).

**Figure 4:**
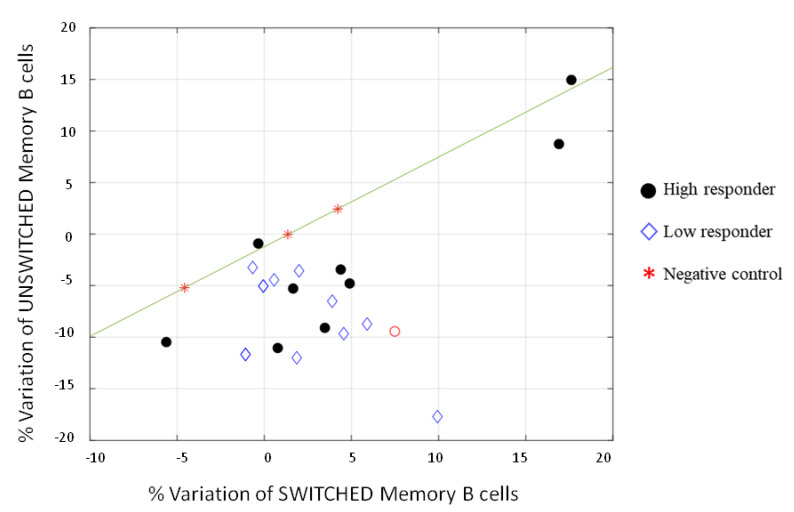
Representation of the relative variation of switched and unswitched memory B-cell populations normalized to the overall amount of B-cells. The mathematical expression y = 0.8694 * x − 1.2207 indicates that any point laying below y is, with high probability, a high or low responder.

We analyzed CD4^+^ central memory (CM CD45RA^−^ CCR7^+^), CD4^+^ effector memory (EM CD45RA^−^ CCR7^−^), CD4^+^ Th17 (CXCR3 CCR6^+^), CD8^+^ central memory (CM CD45RA^−^ CCR7^+^), CD8^+^ effector memory (EM CD45RA^−^ CCR7^−^), naïve B (CD19^+^ CD27^−^ IgD^+^), transitional B (CD19^+^ CD27^−^ IgD^+^ CD24^high^ CD38^high^), unswitched memory B (CD19^+^ CD27^+^ IgD^+^), and switched memory B cells (CD19^+^ CD27^+^ IgD^−^). CD4^+^ and CD8^+^ naïve (CD45RA^+^ CCR7^+^) and effector memory cells re-expressing CD45RA (TEMRA CD45RA^+^ CCR7^−^) together with CD4^+^ Th1 (CXCR3^+^ CCR6^−^) and Th2 (CXCR3^−^ CCR6^−^) helper cells were also analyzed, but no difference was recorded (data not shown). Full gating procedures are provided in Supplementary Materials Figures S1 and S2. As shown in Figure 3, wide variability was observed in T-cell response, indeed low and high responders did not show a relevant difference in behavior. An increasing trend of CD4^+^ and CD8^+^ effector memory cells after Spike stimulation was noted, however, without a significant difference (>0.05). On the contrary, an increase of Th17 cells was surprisingly evidenced upon stimulation with the Spike protein in low and high responders. This subset of CD4^+^ cells produces several effector molecules, including IL-21, which stimulates B-cell differentiation and antibody class switching [12]. This feature correlates with the data clearly shown in Figure 3, where the unswitched memory B-cell differentiated in switched memory cells after exposure to the Spike, indicating that also people with a low neutralizing antibody titer could have a good memory B-cell immune response if reinfected with SARS-CoV-2. This relative variation is better represented in Figure 4, where only two out of twenty responders had a high level of unswitched and switched memory B cells, and one did not show any variations. The remaining samples, both from low and high responders, presented a shift to switched memory B-cells after antigen exposure. One of the controls developed a weak variation in switched memory B cells, which might be attributable to a partial cross-reactive immunity to the common cold human coronaviruses. 

**Figure 5:**
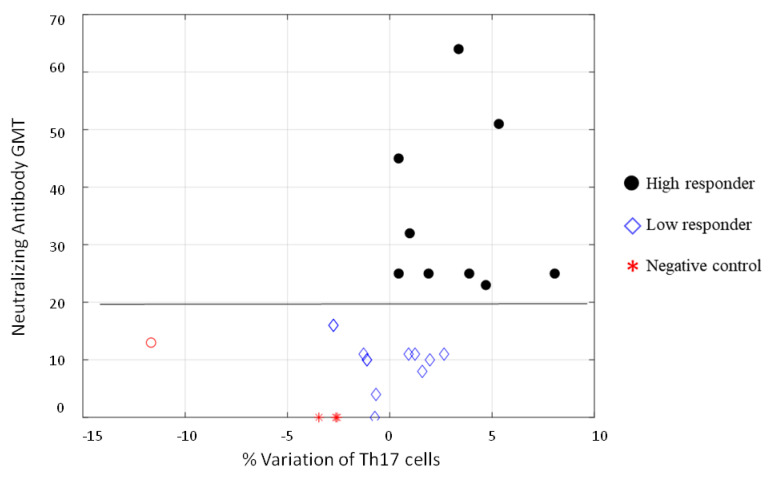
Representation of the % variation after stimulation of the CD4^+^ Th17 population, normalized with respect to total CD4^+^ counted cells, with respect to NT antibody GMT. It is possible to notice that a cut-off can be set with an NT value of 19.5 (calculated average). The red circle was considered an outlier.

Finally, we found an interesting correlation between Th17 cells and neutralizing antibodies to SARS-CoV-2. Figure 5 show that all samples had a positive percentage of Th17 variation corresponded to subjects having neutralizing antibodies; only five low responders (NT antibody titer <20 after one month) and a high responder (red circle in Figure 5) did not present a positive variation of Th17 after six months. A cut-off could be set with an NT value of 19.5.

**Figure 6:**
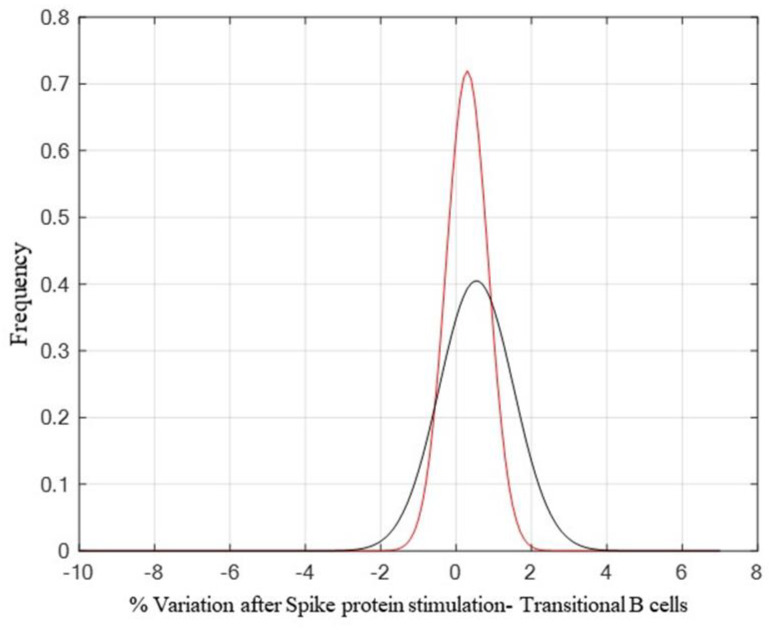
A comparison between the control probability density function (pdf) (*p* = 0.4170 and h0 = 0), shown in red, and the high responder population pdf (*p* = 0.7146 and h0 = 0), shown in black, highlights a small increase after antigen stimulation of transitional B-cells. Tests for the verification of pdf adherence have been conducted through the Chi2 square method.

Moreover, the transitional B-cells showed an increase after Spike stimulation, suggesting their role as developmental intermediates for human mature B-cell generation (Figure 6). We did not include the analysis of naïve and TEMRA cells in Figure 3 since no difference was evidenced between the stimulated and un-stimulated samples.

### 3.3. Fuzzy System

**Figure 7:**
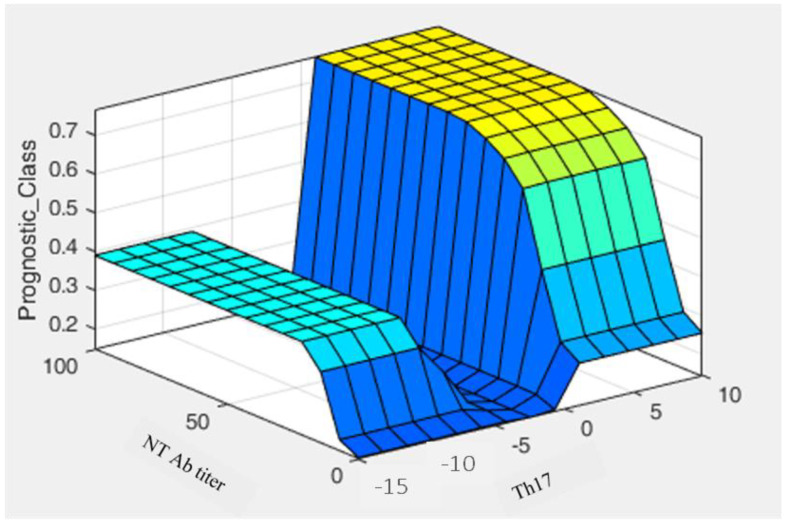
Graphical representation of a decision rule showing neutralizing antibody titer and Th17 population. Fuzzy MATLAB Toolbox and exploited the Mamdani model were used to generate the fuzzy algorithm.

On the basis of these results, we tried to design an automated method based on a fuzzy algorithm that could provide a prognostic tool [13,14]. New computing methods based on fuzzy logic can be used in the development of intelligent systems for decision making, pattern recognition, and control. Figure 7 show the prediction probability of being a high/low responder with respect to an increase of Th17, which is always related to the presence of a good protective antibody response against SARS-CoV-2. The algorithm matches different membership functions designed to describe the likelihood of activating certain probability areas. When both Th17 and NT values are low, the chance to be a high responder is very low and close to zero.

### 3.4. Interferon-γ Production by SARS-CoV-2 Specific T Cells in the Whole Blood

As described in Section 2.6, a technically simple and rapid alternative to Elispot was performed to quantify the amount of interferon-γ in whole blood of the 20 previously selected subjects six months after vaccination, after O/N stimulation with a pool of SARS-CoV-2 peptides derived from the Spike amino acid sequence. A negative control, represented by uninfected and unvaccinated subjects, was included in the test. Table 2 show that 11 out of 20 subjects still had a Spike-specific T cell response, while both the 9 remaining vaccinated subjects and the negative control did not present an interferon-γ production. However, the amount of interferon-γ revealed in the responding subjects was correlated neither with the amount of specific CD4^+^ or CD8^+^ cells obtained by cytofluorimetric analysis nor with their antibody level six months after vaccination.

## 4. Discussion

In this study, the dynamic of the immune response six months after the BNT162b2 mRNA vaccine was analyzed. There are several articles in the scientific literature regarding the immunity after SARS-CoV-2 natural infection [15,16,17,18,19] and on the duration of the immune response after vaccination [4,9,10,20,21]. Here, we assessed the antibody response of 178 HCWs without any history of SARS- CoV-2 infection by chemiluminescent and microneutralization assays 10 days, 1, 3, and 6 months after two doses of the BNT162b2 mRNA vaccine. Only two out of the 53 randomly selected subjects, screened for the presence of specific IgG after the first dose of vaccine, were seronegative (3.8%), while all 178 HCWs were positive 10 days after the second dose. An antibody decline was observed over time, comparing the IgG levels at different time points after the second dose of vaccine.

Similarly, the neutralizing antibody response among the subjects showing no significant difference in GMT 10 (GMT = 109.78) and 30 (GMT = 92.90) days post-vaccination clearly declined in the following months. Indeed, a significant decrease in antibody response was found three (GMT = 32.4; *p* < 0.0001 vs. 30 days) and six months (GMT = 19.4; *p* < 0.0001 vs. three months) after vaccination (Figure 1).The NT antibody titer reached an average of 19.4, likely representing the cut-off to confer protection against the Wuhan strain of SARS-CoV-2 (19). Interestingly, we noticed that vaccinated subjects having a GMT > 128 10 days after the vaccine showed a slight decrease of the titer 30 days after, while those having a GMT < 128 showed quite different behavior. Most of them (56.6%) developed a titer increase, a few (23.3%) showed a slight decrease, the remaining (20%) maintained the same titer (Table 1). However, it appeared that a levelling of the neutralizing antibody titer occurred over time, with a decreasing titer in subjects with a high amount of IgG and a titer increase in those showing a low–modest IgG level. Neutralizing antibodies are considered a good correlate of protection [22,23,24], and B-cells participate in the antiviral immune response. We stimulated human PBMCs with the Spike protein in vitro in order to understand whether a possible contact with SARS-CoV-2 in a vaccinated subject could mount an adequate immune response to protect the host in case of reinfection. After vaccination, memory B-cells capable of responding to a challenge were produced and distinguished in different subsets. This kind of response was present in all analyzed subjects, both high and low responders. We noticed that unswitched memory B-cells (IgD^+^) shifted to switched memory B-cells (IgD^−^) after in vitro stimulation with the Spike, indicating that the immune system is ready to defend the host in case of virus attack. Indeed, memory B cells play an important role in SARS-CoV-2 immunity and provide a good indication of vaccine efficacy [25,26]. While naïve B-cells showed a variable profile, transitional B-cells demonstrated a modest increase after antigen stimulation, particularly in high responders (Figure 6). Transitional B-cell subsets, identified within the CD24^high^CD38^high^ B population display differential regulatory abilities. A subset of them is specialized in suppressing the production of proinflammatory cytokines and has the capacity to produce high levels of IL-10, which enhances B-cell survival, proliferation, and antibody production, thus providing support to the reported increase of memory B-cells. However, the biology of transitional B-cells still remains controversial [1]. Finally, we found a good correlation between Th17 cells after Spike stimulation and the presence of neutralizing antibodies. Th17 cells have a role in supporting B-cell responses. Indeed, under polarizing conditions, these cells can activate T-cells to support the expansion of B-cells and an enhanced antibody response [12,27,28]. In this study, we found that all high responders had an increase of Th17 cells after the exposure to the antigen, but only half of the low responders presented a similar behavior [22]. Thus, analysis of Th17 could be considered a useful biomarker correlated with an effective neutralizing antibody response for evaluating the efficacy of the vaccine over time. To this aim, we also implemented a prognostic tool based on fuzzy logic and artificial intelligence designed for this specific control task. By applying fuzzy logic, we were able to confirm this result. Indeed, in real life, things are not either white or black, but most are grey, thus in many practical situations, it is convenient to consider intermediate logical values, such as in these biological events that show variability among individuals. Fuzzy logic by intrinsic nature is not crisp, and therefore it weights, by means of specific membership function, the influence of several factors such as the Th17 and NT antibody titer amount for classification based on *a priori* knowledge.

Analyzing the virus-specific CD4^+^ and CD8^+^ T-cell immune response six months after vaccination, we noticed a variable profile both for CD4^+^ and CD8^+^ central memory lymphocytes, with a modest but not significant (*p* > 0.05) increase of the specific effector memory cells. Only 35% and 40% of the subjects revealed a 1% increase of CD4^+^ and CD8^+^ cells, respectively. We observed a clear heterogeneity of the T-cell-mediated response after BNT162b vaccination. These data were also supported by the low expression of interferon-γ non-correlated with the induced Spike cellular response in vaccinated subjects.

These data provide evidence that despite the antibody decline six months after BNT162b2 mRNA vaccination, the memory B-cell persists, and transitional B-cells could have a role together with Th17 cells in the proliferation and differentiation of B-lymphocytes. On the contrary, in this limited number of cases, a heterogeneity of CD4^+^ and CD8^+^ effector memory cell response to the Spike protein stimulation leads to hypothesize that in particular CD8+ cells do not represent the first class of defense against SARS-CoV-2 six months after mRNA vaccination. Humoral response against SARS-CoV-2 represents a valid correlate of protection; however, the T-cell response is also important, particularly for those who have a low antibody response and need to compensate for this shortage. Therefore, estimation of immunity over time is fundamental to improve the vaccine, evaluate the variables that are useful to determine the real need for other boosts to protect people, and possibly develop an adequate time schedule of vaccination.

## Figures and Tables

**Figure 2:**
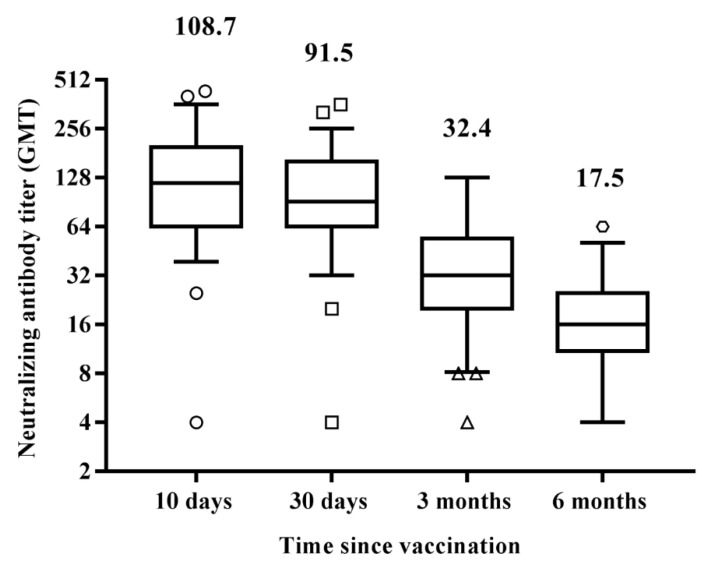
Differences in neutralizing antibody titers (GMT) among the different time samplings after receiving the second vaccine administration. Results are reported in the box-whisker plots as median GMT and upper and lower quartiles. GMT, geometric mean titer. Differences in antibody titers were evaluated, and the statistical significance was assessed using the two-tailed chi-square test. Results were considered statistically significant at *p* < 0.05. Analyses were performed by using Graph Pad Prism software (v.7.0).

**Table 1:** Neutralizing antibody profiles 1, 3 and 6 months after the second dose of vaccination in 60 subjects compared with the antibody response developed at 10 days.

	Neutralizing Antibody GMT > 128 at 10 Days Post-Vaccination			Neutralizing Antibody GMT < 128 at 10 Days Post-Vaccination		
	1 Month	3 Months	6 Months	1 Month	3 Months	6 Months
Ab level increase average	2 (6.66%) + 21.7%	0	0	17 (56.66%) + 22.0%	0	0
Ab level stability	2 (6.66%)	0	0	6 (20.0%)	0	0
Ab level decrease average	26 (86.66%) − 28.6%	30 (100%) − 70.7%	30 (100%) − 86.5%	7 (23.33%) − 6.8%	30 (100%) − 58.7%	30 (100%) − 76.9%

**Table 2:** The table shows the CoviFERON FIA results of the 20 samples previously analyzed by multiparametric flow cytofluorimetry.

N	CoviFERON FIA Results (IU/mL)				
	Nil	Original SP Antigen	Mitogen	Results Interpretation	
1	<0.145	1.42	>10.00	1.28	Reactive
2	<0.145	4.55	>10.00	4.41	Reactive
3	<0.145	1.12	>10.00	0.98	Reactive
4	<0.145	0.17	>10.00	0.03	Non-Reactive
5	<0.145	1.76	>10.00	1.62	Reactive
6	<0.145	0.93	>10.00	0.79	Reactive
7	<0.145	0.24	>10.00	0.10	Non-Reactive
8	<0.145	<0.145	>10.00	0.00	Non-Reactive
9	2.38	2.23	>10.00	−0.15	Non-Reactive
10	<0.145	0.56	>10.00	0.42	Reactive
11	<0.145	1.34	>10.00	1.20	Reactive
12	<0.145	<0.145	>10.00	0.00	Non-Reactive
13	<0.145	1.08	>10.00	0.94	Reactive
14	<0.145	2.74	>10.00	2.60	Reactive
15	<0.145	1.17	>10.00	1.03	Reactive
16	0.63	0.89	>10.00	0.26	Non-Reactive
17	0.27	7.00	>10.00	6.73	Reactive
18	<0.145	0.22	>10.00	0.08	Non-Reactive
19	<0.145	<0.145	>10.00	0.00	Non-Reactive
20	<0.145	0.18	>10.00	0.04	Non-Reactive
CTRL	<0.145	<0.145	>10.00	0.00	Non-Reactive

## Data Availability

Further informations about data supporting the reported results, should be directed and will be fulfilled by the lead contact Maria Grazia Cusi (mariagrazia.cusi@unisi.it), upon reasonable request.

## Supplementary Materials

The following supporting information can be downloaded at: https://www.mdpi.com/article/10.3390/vaccines10020171/s1, Figure S1: Representative gating strategies for CD4^+^ and CD8^+^ cell populations by multiparametric flow cytometry, Figure S2: Representative gating strategies for CD19^+^, CD20^+^ B cell populations by multiparametric flow ccytometry.
