# Supplementary material for: Overview of Anti-SARS-CoV-2 Immune Response Six Months after BNT162b2 mRNA Vaccine
Source: Vaccines (Basel). 2022 Jan 22;10(2):171. doi: 10.3390/vaccines10020171 (PMC8879995; doi:10.3390/vaccines10020171)
Supplement: Supplementary file 1 [file vaccines-10-00171-s001.zip › vaccines-1538672-supplementary.pdf]

Supplementary Materials

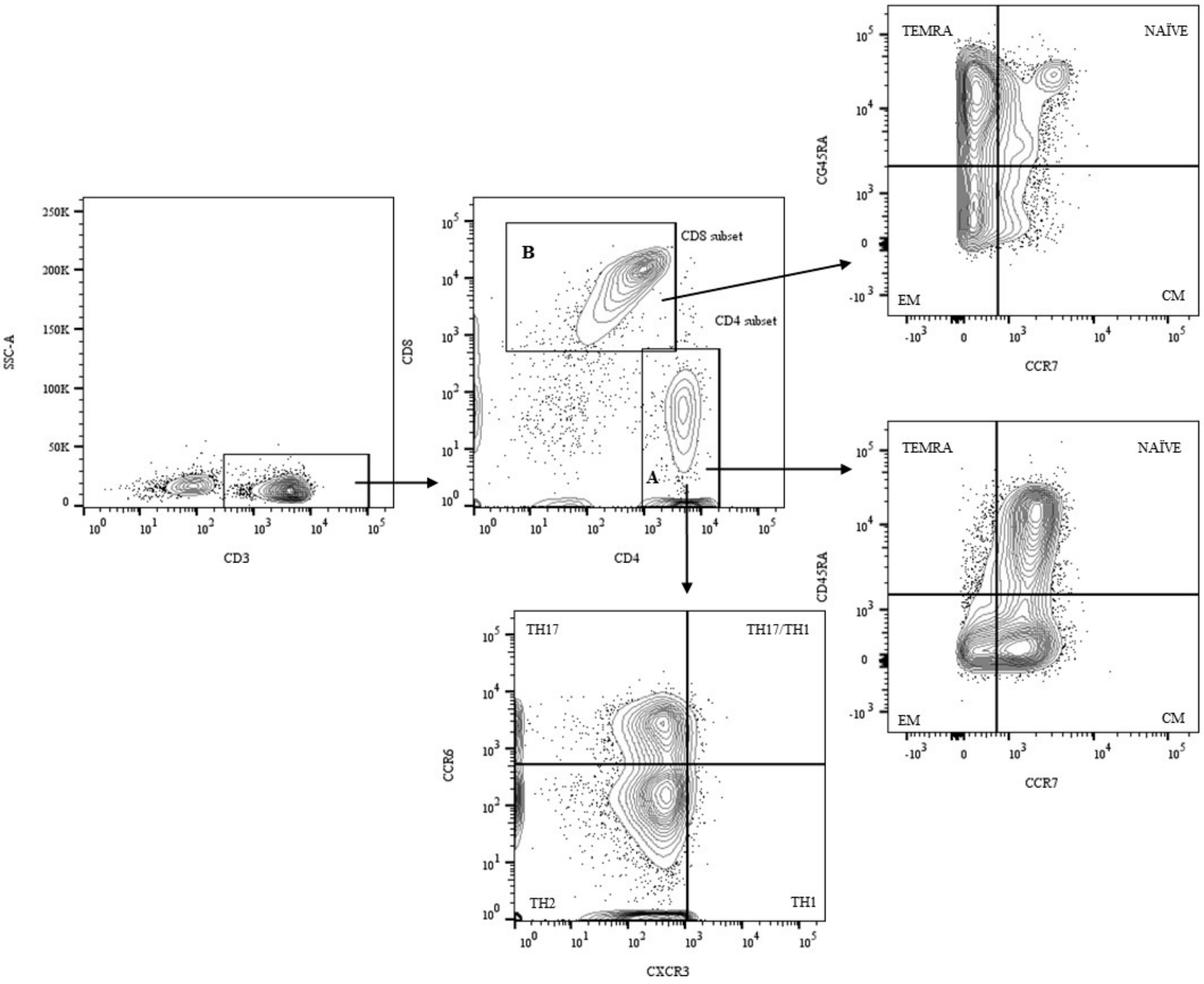

Figure S1. Representative gating strategies for $CD4^+$ and $CD8^+$ T cell populations by multiparametric flow cytometry

A. Identification of memory T cells and Th1, Th2, and Th17 helper populations inside the $CD3^+$ $CD4^+$ cell subset: Central Memory (CM $CD45RA^-$ $CCR7^+$), Effector Memory (EM $CD45RA^-$ $CCR7^-$), Effector Memory cells re-expressing CD45RA (TEMRA $CD45RA^+$ $CCR7^-$), Naïve ($CD45RA^+$ $CCR7^+$); Th17 ($CXCR3^-$ $CCR6^+$), Th17/Th1 ($CXCR3^+$ $CCR6^+$) Th1 ($CXCR3^+$ $CCR6^-$), Th2 ($CXCR3^-$ $CCR6^-$) helper cells. **B.** Identification of memory T populations inside the $CD3^+$ $CD8^+$ cell subset: Central Memory (CM $CD45RA^-$ $CCR7^+$), Effector Memory (EM $CD45RA^-$ $CCR7^-$), Effector Memory cells re-expressing CD45RA (TEMRA $CD45RA^+$ $CCR7^-$), Naïve ($CD45RA^+$ $CCR7^+$).

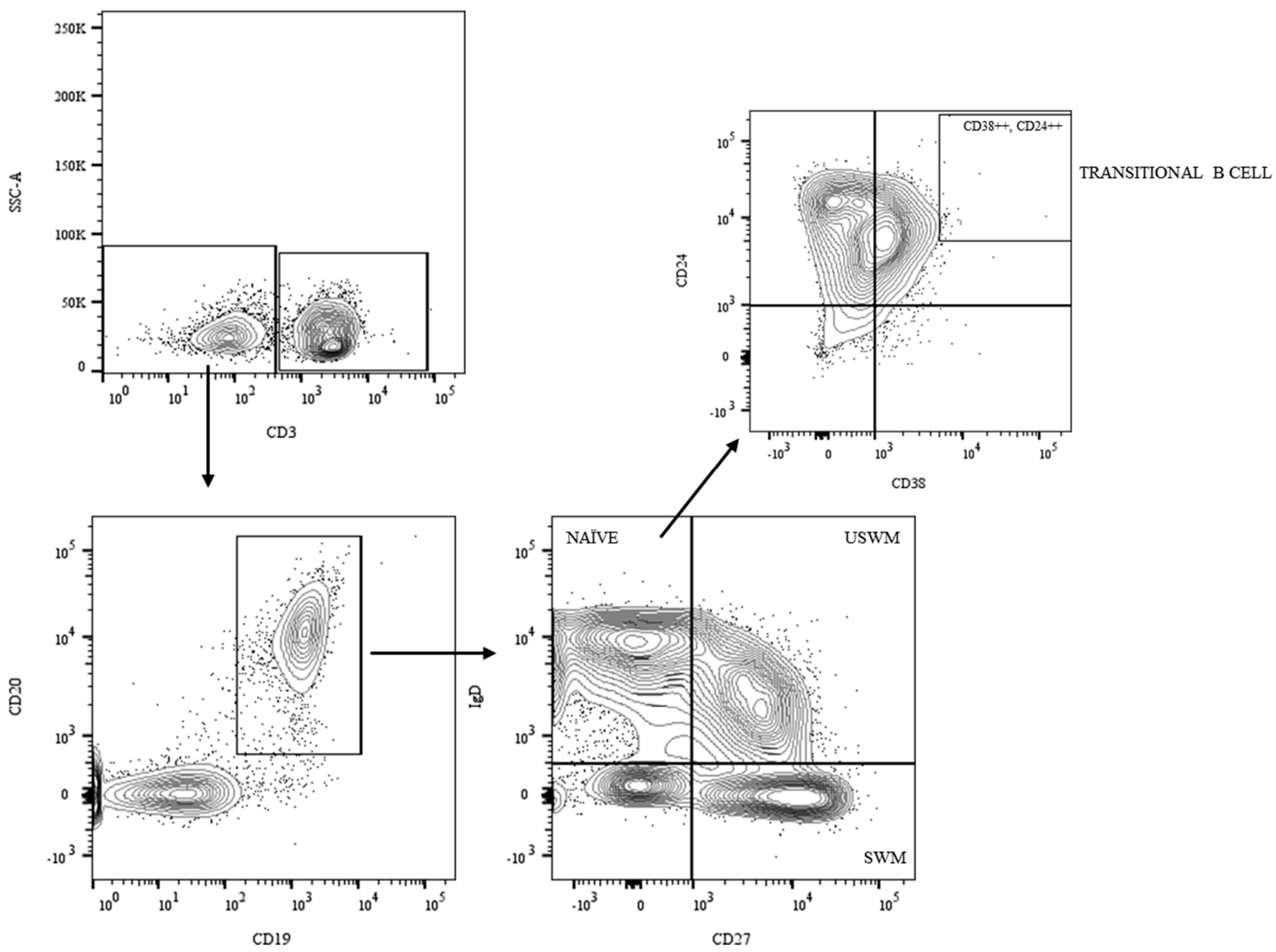

Figure S2. Representative gating strategies for C19+, CD20+ B cell populations by multiparametric flow cytometry

Characterization of B cell populations inside the CD3- CD19+CD20+ cell gate: Naïve (CD19+ CD27- IgD+), Transitional (CD19+ CD27- IgD+ CD24high CD38high), Unswitched Memory (CD19+ CD27+ IgD+) and Switched Memory (CD19+ CD27+ IgD-) cells.
